# Canagliflozin and Heart Failure in Type 2 Diabetes Mellitus: Results From the CANVAS Program

**DOI:** 10.1161/CIRCULATIONAHA.118.034222

**Published:** 2018-07-30

**Authors:** Karin Rådholm, Gemma Figtree, Vlado Perkovic, Scott D. Solomon, Kenneth W. Mahaffey, Dick de Zeeuw, Greg Fulcher, Terrance D. Barrett, Wayne Shaw, Mehul Desai, David R. Matthews, Bruce Neal

**Affiliations:** 1 Department of Medicine and Health Sciences, Division of Community Medicine, Primary Care, Faculty of Health Sciences, Department of Local Care West, County Council of Ötergötland, Linköping University, Sweden (K.R.).; 2 The George Institute for Global Health (K.R., V.P., B.N.); 3 Faculty of Medicine (B.N.); 4 University of New South Wales, Sydney, Australia. Royal North Shore Hospital (G.F., V.P., G.F.); 5 Charles Perkins Centre (B.N.); 6 University of Sydney, Australia. Harvard Medical School and Brigham and Women’s Hospital, Boston, MA (S.D.S.).; 7 Department of Medicine, Stanford Center for Clinical Research, Stanford University School of Medicine, CA (K.W.M.).; 8 University of Groningen, University Medical Center Groningen, The Netherlands (D.d.Z.).; 9 Janssen Research & Development, LLC, Raritan, NJ (T.D.B., W.S., M.D.).; 10 Oxford Centre for Diabetes, Endocrinology, and Metabolism, University of Oxford, United Kingdom (D.R.M).; 11 Harris Manchester College, University of Oxford, United Kingdom (D.R.M.).; 12 Imperial College London, United Kingdom (B.N.).

**Keywords:** canagliflozin, heart failure, randomized trial, SGLT2 inhibitor, type 2 diabetes mellitus

## Abstract

**Background:**

Canagliflozin is a sodium glucose cotransporter 2 inhibitor that reduces the risk of cardiovascular events. We report the effects on heart failure (HF) and cardiovascular death overall, in those with and without a baseline history of HF, and in other participant subgroups.

**Methods:**

The CANVAS Program (Canagliflozin Cardiovascular Assessment Study) enrolled 10 142 participants with type 2 diabetes mellitus and high cardiovascular risk. Participants were randomly assigned to canagliflozin or placebo and followed for a mean of 188 weeks. The primary end point for these analyses was adjudicated cardiovascular death or hospitalized HF.

**Results:**

Participants with a history of HF at baseline (14.4%) were more frequently women, white, and hypertensive and had a history of prior cardiovascular disease (all *P*<0.001). Greater proportions of these patients were using therapies such as blockers of the renin angiotensin aldosterone system, diuretics, and β-blockers at baseline (all *P*<0.001). Overall, cardiovascular death or hospitalized HF was reduced in those treated with canagliflozin compared with placebo (16.3 versus 20.8 per 1000 patient-years; hazard ratio [HR], 0.78; 95% confidence interval [CI], 0.67–0.91), as was fatal or hospitalized HF (HR, 0.70; 95% CI, 0.55–0.89) and hospitalized HF alone (HR, 0.67; 95% CI, 0.52–0.87). The benefit on cardiovascular death or hospitalized HF may be greater in patients with a prior history of HF (HR, 0.61; 95% CI, 0.46–0.80) compared with those without HF at baseline (HR, 0.87; 95% CI, 0.72–1.06; *P* interaction =0.021). The effects of canagliflozin compared with placebo on other cardiovascular outcomes and key safety outcomes were similar in participants with and without HF at baseline (all interaction *P* values >0.130), except for a possibly reduced absolute rate of events attributable to osmotic diuresis among those with a prior history of HF (*P*=0.03).

**Conclusions:**

In patients with type 2 diabetes mellitus and an elevated risk of cardiovascular disease, canagliflozin reduced the risk of cardiovascular death or hospitalized HF across a broad range of different patient subgroups. Benefits may be greater in those with a history of HF at baseline.

**Clinical Trial Registration:**

URL: https://www.clinicaltrials.gov. Unique identifiers: NCT01032629 and NCT01989754.

## Abstract 2

Supplemental Digital Content is available in the text.

**Clinical Perspective**

**What Is New?**

- The sodium glucose cotransporter 2 inhibitor canagliflozin reduced the risk of a range of composite and cause-specific heart failure (HF) outcomes.
- Benefits from canagliflozin may be greater in those with a history of HF.
- There was no evidence that patients with a history of HF were likely to suffer higher rates of adverse events from canagliflozin.

**What Are the Clinical Implications?**

- Patients with type 2 diabetes mellitus at risk of HF are particularly likely to benefit from treatment with canagliflozin.
- Beneficial effects of canagliflozin on HF outcomes are likely to be accrued on top of other therapies for HF management.

Type 2 diabetes mellitus is associated with a substantial risk of cardiovascular and renal disease, including heart failure (HF).^1–3^ HF in diabetes mellitus is attributed to macrovascular and microvascular dysfunction, volume overload, impaired renal function, and direct effects of diabetes mellitus and insulin resistance on cardiac myocytes.^4–7^ Mortality outcomes for patients with type 2 diabetes mellitus and HF are worse than for patients with either of the diseases alone, with a median survival of just 4 years.^8^ Before the introduction of sodium glucose cotransporter 2 (SGLT2) inhibitors, treatment with glucose-lowering agents has not been shown to reduce HF hospitalization,^9^ and there is evidence of increased risks of HF in some trials of dipeptidyl peptidase-4 inhibitors^10,11^ and the thiazolidinedione class.^9^ Two landmark clinical trials using inhibitors of SGLT2—EMPA-REG OUTCOME^12^ and the CANVAS Program (Canagliflozin Cardiovascular Assessment Study)^13^—have demonstrated reductions in the risk of hospitalization for HF, with benefits of empagliflozin reported across a broad range of patient groups.^14^ The present analyses explored in further detail the effects of canagliflozin on HF and determined the effects of canagliflozin on a range of efficacy and safety outcomes among CANVAS Program participants with and without a history of HF at baseline.

## Methods

### Program Design

The study design, characteristics of participants, and main results of the CANVAS Program have previously been published.^13,15^ In brief, the CANVAS Program, comprising the 2 similarly designed and conducted trials, CANVAS and CANVAS-R (CANVAS-Renal), was designed to assess the cardiovascular and renal safety and efficacy of canagliflozin compared with placebo, and also assess how any potential benefits might balance against risks. In total, 667 centers in 30 countries were involved in the 2 trials that were scheduled for joint closeout and analysis when ≥688 cardiovascular events and ≥78 weeks of follow-up had been accrued for the last randomized participant, which occurred in February 2017. A complete list of investigators and committees in the CANVAS Program is provided in the Appendix in the online-only Data Supplement. Data from the CANVAS Program will be made available in the public domain via the Yale University Open Data Access Project (http://yoda.yale.edu/) once the product and relevant indication studied have been approved by regulators in the United States and European Union and the study has been completed for 18 months. The trial protocols and statistical analysis plans were published along with the primary CANVAS Program article.^13^

### Participants

Participants included in the CANVAS Program were men and women with type 2 diabetes mellitus (glycohemoglobin ≥7.0% and ≤10.5% and estimated glomerular filtration rate >30 mL/min/1.73 m^2^). Participants were also required to be either ≥30 years of age with a history of symptomatic atherosclerotic cardiovascular disease or ≥50 years of age with ≥2 risk factors for cardiovascular disease (duration of diabetes mellitus ≥10 years, systolic blood pressure >140 mm Hg while on ≥1 antihypertensive agents, current smoker, documented microalbuminuria or macroalbuminuria, or documented high-density lipoprotein cholesterol <1 mmol/L). Patients with New York Association Class IV HF were excluded. The definition of HF at baseline was based on physician review of the patient’s medical history at the first visit, with no requirement for collection of diagnostic biomarkers or the conduct of echocardiography. All participants provided informed consent, and ethics approval was obtained for every center.

### Randomization, Treatment, and Follow-Up

After a 2-week, single-blind, placebo run-in period, participants were randomized centrally through an interactive web response system using a computer-generated randomization schedule prepared by the study sponsor using randomly permuted blocks. Participants in CANVAS were assigned in a 1:1:1 ratio to canagliflozin 300 mg, canagliflozin 100 mg, or matching placebo, and participants in CANVAS-R were randomly assigned in a 1:1 ratio to canagliflozin or matching placebo, administered at an initial dose of 100 mg daily with optional uptitration to 300 mg from week 13. Participants and all study and sponsor staff were masked to individual treatment allocations until the completion of the study. Use of other background therapy for glycemic management, treatment of HF, and other risk factor control was according to best practices instituted in line with local guidelines.

Participants were followed after randomization in a face-to-face follow-up that was scheduled for 3 visits in the first year and at 6-month intervals thereafter, with alternating telephone follow-up between face-to-face assessments. Every follow-up included inquiry about primary and secondary outcome events and serious adverse events. Serum creatinine measurement with estimated glomerular filtration rate was performed at least every 26 weeks in both trials. Participants who prematurely discontinued study treatment continued scheduled follow-up wherever possible, with extensive efforts made to obtain full outcome data for all participants during the final follow-up window that spanned from November 2016 to February 2017.

### Outcomes

The primary outcome for these analyses was the composite of cardiovascular death or hospitalized HF. The detailed criteria used to define outcomes are included in the Appendix in the online-only Data Supplement. Cardiovascular death included death resulting from an acute myocardial infarction, sudden cardiac death, death because of HF, death because of stroke, and death because of other cardiovascular causes. Hospitalized HF was an event that required an admission to an inpatient unit or a visit to an emergency department, resulting in a ≥24-hour stay and ≥1 clinical symptoms of worsening HF, ≥2 physical signs of HF and a need for additional or increased therapy, and the absence of other noncardiac etiology or other cardiac etiology that might explain the presentation.

Secondary outcomes were fatal or hospitalized HF, fatal HF, hospitalized HF, the composite of major adverse cardiovascular events (cardiovascular death, nonfatal myocardial infarction, and nonfatal stroke), fatal or nonfatal myocardial infarction, fatal or nonfatal stroke, all-cause mortality, and serious decline in kidney function (defined as a composite of 40% reduction in estimated glomerular filtration rate sustained for ≥2 consecutive measures, the need for renal replacement therapy, or death from renal causes). The safety outcomes assessed were all serious adverse events and all adverse events leading to discontinuation, as well as amputation, fracture, osmotic diuresis–related adverse events (according to the Medical Dictionary for Regulatory Activities preferred terms: increase in urine output such as polyuria, pollakiuria, micturition urgency and nocturia, as well as those related to thirst; polydipsia, dry mouth, throat dry, or tongue dry), and volume depletion–related adverse events. End point adjudication committees adjudicated all cardiovascular outcomes, renal outcomes, deaths, and fractures. Fatal HF events were those with HF adjudicated as the proximate cause of death.

### Statistical Analysis

Categorical variables were summarized as the number of patients with corresponding percentages, and continuous variables were summarized as the mean and standard deviation. Differences in baseline characteristics between participants with a history of HF compared with participants with no history of HF were evaluated using a χ^2^ test for categorical variables, a *t* test for continuous normally distributed variables, and a Wilcoxon 2-sample test for continuous variables with a skewed distribution (distributions were evaluated using an Anderson–Darling test).

Efficacy analyses were based on the full integrated dataset and the intent-to-treat approach, with the comparison being between all participants assigned to canagliflozin (regardless of dose) and all participants assigned to placebo. Annualized incidence rates per 1000 patient-years of follow-up were calculated for all outcomes in addition to hazard ratios (HRs) and 95% confidence intervals (CIs) determined from Cox regression models that included a trial stratification factor. Absolute risk differences for 1000 patients over 5 years and corresponding 95% CIs were estimated as the differences in the incidence rates between randomized treatment groups using a Poisson regression analysis with an assumption of constant annual event probabilities.^16^ On-treatment analysis (based on patients who experienced a safety outcome while on study drug or in ≤30 days of study drug discontinuation) was used for the safety outcomes, except for amputation and fracture, which were assessed using intent-to-treat analyses. For all outcome analyses, we tested the homogeneity of treatment effects across the 2 contributing trials using *P* values for interactions based on the joint test in the Cox regression models, and the same approach was used for testing comparability of effects across subgroups defined by baseline participant characteristics. There was no formal statistical adjustment for multiple comparisons, and *P* values were interpreted in light of the many assessments made. Analysis of recurrent hospitalization for HF was assessed with an Andersen–Gill model. Analyses were performed using SAS version 9.2, SAS Enterprise Guide version 7.1, and STATA version 13.1.

## Results

There were 10 142 patients with type 2 diabetes mellitus in the CANVAS Program, and the mean follow-up time was 188.2 weeks. Mean age was 63.3 years, 35.8% of participants were women, the mean duration of diabetes mellitus was 13.5 years, and 65.6% had a history of cardiovascular disease. In addition, 1461 (14.4%) participants reported a history of HF at baseline. These participants were significantly different from the remaining participants in most aspects of demographics and disease history, in addition to exhibiting greater use of concomitant therapies used for the management of HF, including diuretics, renin angiotensin aldosterone system blockers, and β-blockers, but lower usage of statins and metformin (all *P*<0.001; Table). There were 203 cardiovascular deaths or hospitalized HF events recorded among those participants who reported a history of HF at baseline and 449 among those who did not.

**Table.**
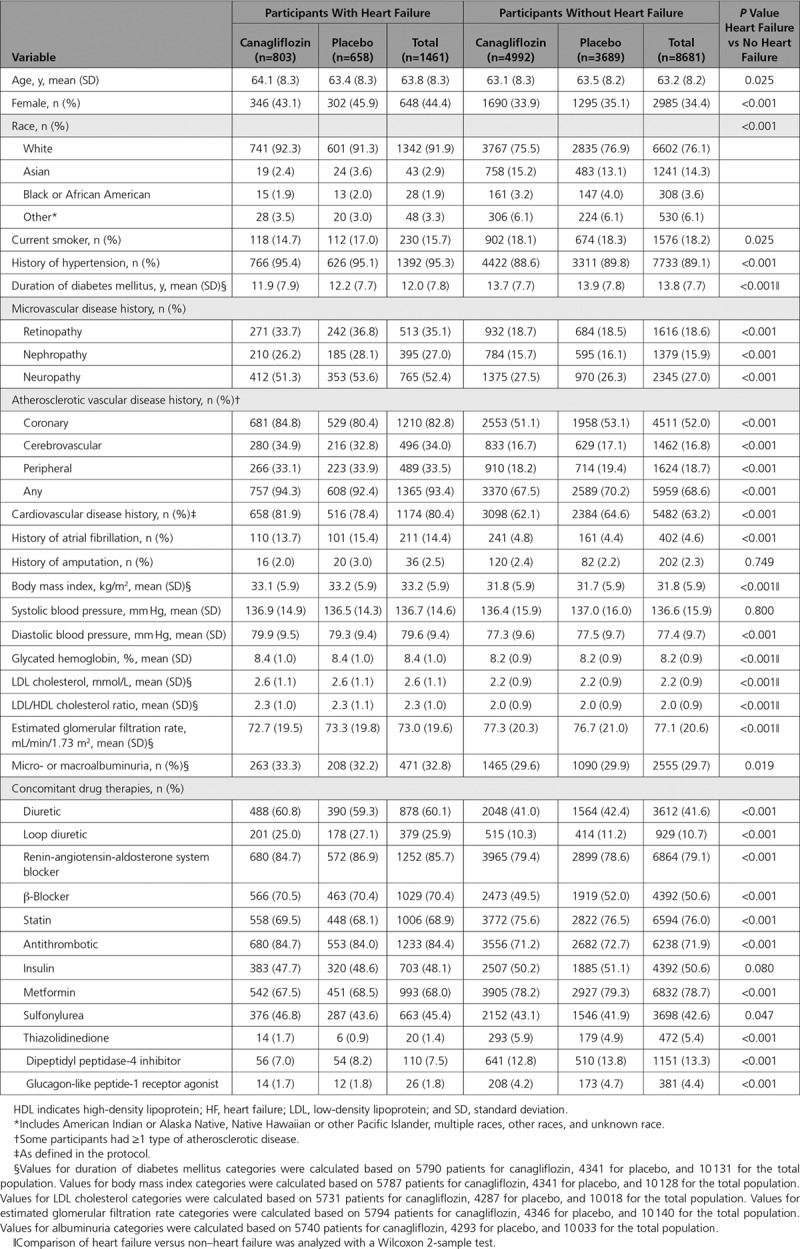
Baseline Characteristics of Participants With and Without Heart Failure at Baseline

### Effects of Canagliflozin on HF Outcomes (Overall and in Patient Subgroups)

**Figure 1.**
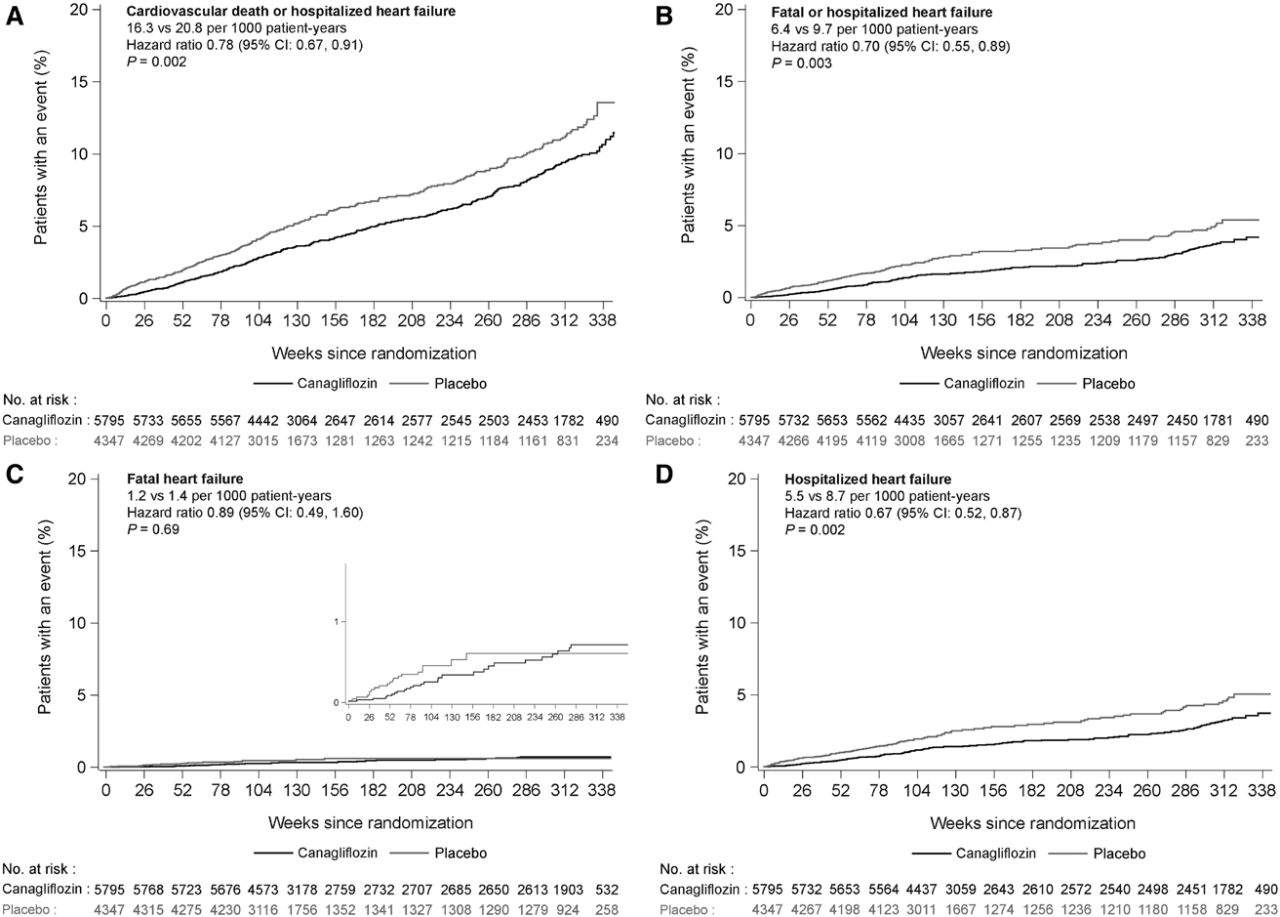
**Effects of canagliflozin on heart failure outcomes. A** through **D**, Effects of canagliflozin on cardiovascular death or hospitalized heart failure (**A**), fatal or hospitalized heart failure (**B**), fatal heart failure (**C**), and hospitalized heart failure (**D**). CI indicates confidence interval; and HF, heart failure.

**Figure 2.**
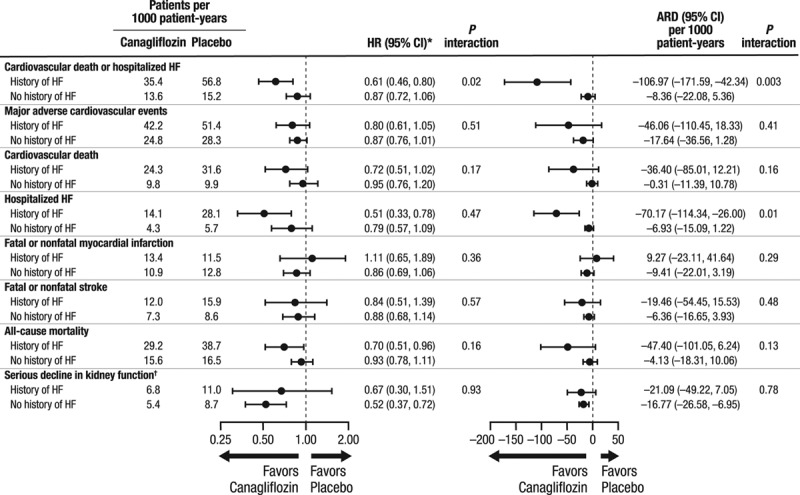
**Proportional and absolute effects of canagliflozin compared with placebo on cardiovascular and renal outcomes in patients with and without a history of heart failure at baseline.** *HR (canagliflozin compared to placebo) and its 95% CI are estimated using a Cox proportional hazard model including treatment as the explanatory variable. The model for CV death is stratified by prior CV disease subgroup and study. The models of renal endpoints are stratified for stage of baseline chronic kidney disease, measured by estimated glomerular filtration rate (<60, ≥60 mL/min/1.73 m^2^) and by study. †Serious decline in kidney function was defined as a 40% reduction in the estimated glomerular filtration rate, the need for renal replacement therapy, or death from renal causes. ARD indicates absolute risk difference over 5 years; CI, confidence interval; HF, heart failure; and HR, hazard ratio.

Compared with placebo, canagliflozin was associated with significantly lower risks of cardiovascular death or hospitalized HF (HR, 0.78; 95% CI, 0.67–0.91), fatal or hospitalized HF (HR, 0.70; 95% CI, 0.55–0.89), as well as hospitalized HF alone (HR, 0.67; 95% CI, 0.52–0.87). There was no clear separate effect on fatal HF (HR, 0.89; 95% CI, 0.49–1.60) for which there were few events and wide CIs (Figure 1). A subsidiary analysis of the primary outcome that accounted for competing mortality resulted in an HR estimate of 0.66 (95% CI, 0.51–0.84). The benefit on cardiovascular death or hospitalized HF was borderline significantly (*P* interaction =0.021) greater in patients with a prior history of HF (HR, 0.61; 95% CI, 0.46–0.80) compared with those without HF at baseline (HR, 0.87; 95% CI, 0.72–1.06; Figure 2). The absolute risk differences were –106.97 (95% CI, –171.59 to –42.34) per 1000 patient-years for participants with a history of HF at baseline and –8.36 (95% CI, –22.08 to 5.36) per 1000 patient-years for participants without a history of HF at baseline (*P* interaction =0.003).

**Figure 3.**
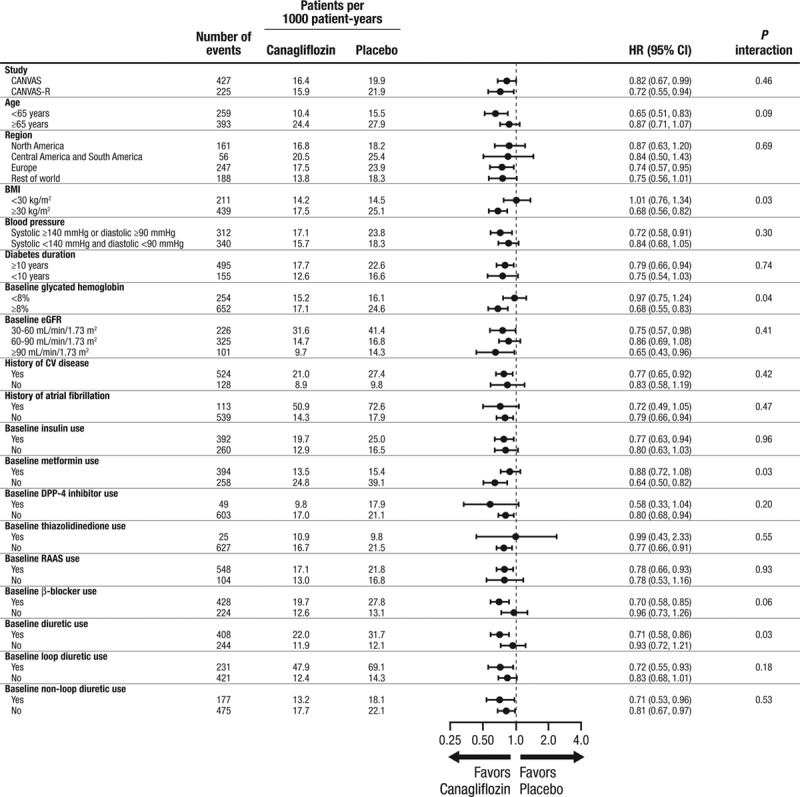
**Effects on cardiovascular death or hospitalized heart failure in subgroups defined by demographic and disease characteristics.** History of CV disease–yes indicates patients were included on the basis of atherosclerotic cardiovascular disease history, whereas history of CV disease–no indicates patients were included on the basis of risk factors alone. BMI indicates body mass index; CANVAS, Canagliflozin Cardiovascular Assessment Study; CANVAS-R, Canagliflozin Cardiovascular Assessment Study–Renal; CI, confidence interval; CV, cardiovascular; DPP-4, dipeptidyl peptidase-4; eGFR, estimated glomerular filtration rate; HR, hazard ratio; and RAAS, renin angiotensin aldosterone system.

Rates of HF varied according to baseline characteristics such as age, renal function, and other disease history characteristics, but effects of canagliflozin on cardiovascular death or hospitalized HF were mostly comparable across participant subgroups (Figure 3). Nominally significant interaction was observed with respect to the cardiovascular death or hospitalized HF outcome for several subgroups, including patients with higher versus lower body mass index, lower versus higher baseline glycohemoglobin, with versus without background use of diuretic therapy, and with versus without background metformin use (all *P* interaction >0.02; Figure 3). Participants randomized to canagliflozin treatment had less recurrent hospitalizations for HF during follow-up compared with participants assigned to placebo (HR, 0.68; 95% CI, 0.47–0.96). In the CANVAS trial, in which participants were assigned at random to placebo, canagliflozin 100 mg, or canagliflozin 300 mg, there was no evidence that the effects on cardiovascular death or hospitalized HF varied by dose (100 mg versus placebo: HR, 0.82; 95% CI, 0.65–1.03; and 300 mg versus placebo: HR, 0.82; 95% CI, 0.65–1.03). Among the subset of participants who reported a history of HF and loop diuretic use at baseline (n=379), the HR for the primary outcome was 0.54 (95% CI, 0.37–0.78).

### Effects of Canagliflozin on Cardiovascular, Kidney, and Death Outcomes in Patients With and Without HF at Baseline

Proportional effects of canagliflozin compared with placebo were comparable in patients with and without HF at baseline for major adverse cardiovascular events, cardiovascular death, myocardial infarction, stroke, all-cause mortality, and serious decline in kidney function (all *P* interaction >0.160; Figure 2). Patients with a history of HF were at higher absolute risk of most outcomes. Although the numeric values for risk differences were typically greater among participants with a history of HF compared with those without, none reached statistical significance (all *P* interaction >0.130).

### Safety Outcomes

**Figure 4.**
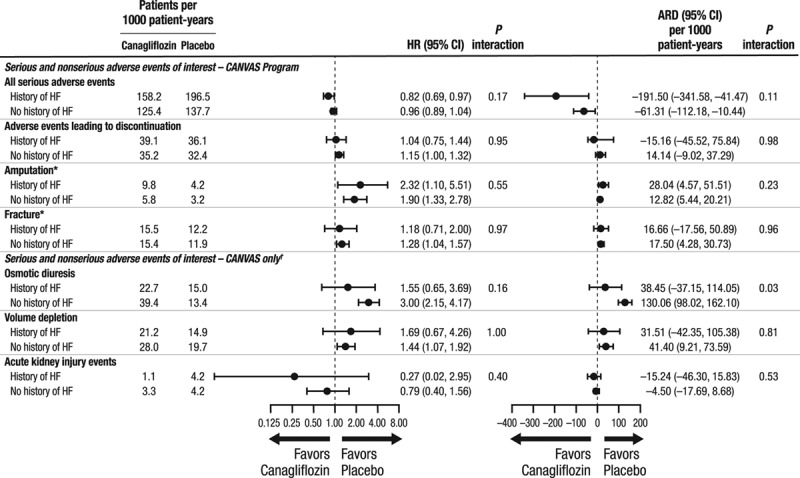
**Proportional and absolute effects of canagliflozin compared with placebo on key safety outcome in patients with and without a history of heart failure at baseline.** *Based on ITT dataset, whereas all other analyses based on on-treatment dataset. †For these adverse events, the annualized incidence rates are reported based on the CANVAS study alone through January 7, 2014, because, after this time, only serious adverse events or adverse events leading to discontinuation were collected. In the CANVAS-R study, only serious adverse events or adverse events leading to discontinuation were collected for these events. ARD indicates absolute risk difference over 5 years; CANVAS, Canagliflozin Cardiovascular Assessment Study; ITT, intent-totreat; CANVAS-R, Canagliflozin Cardiovascular Assessment Study–Renal; CI, confidence interval; HF, heart failure; and HR, hazard ratio.

Compared with placebo, canagliflozin has established associations with increased risks of amputation, fracture, and volume depletion, but there was no evidence of proportional differences in these risks between patients with and without HF at baseline (all *P* interaction >0.160; Figure 4). The absolute risk of osmotic diuresis-related events, another established risk of therapy, was significantly lower in patients with a history of HF compared with those without (*P* interaction =0.029; Figure 4).

## Discussion

Patients with type 2 diabetes mellitus and established cardiovascular disease or at high risk of cardiovascular events who were treated with canagliflozin experienced significantly reduced rates of cardiovascular death or hospitalized HF. Benefits may be greater in those with a history of HF compared with those without. Effects were apparent across a broad range of participant subgroups, including those using established treatments for the prevention of HF, such as blockade of the renin angiotensin aldosterone system, diuretics, and β-blockers.

Other cardiovascular outcomes and death generally occurred more frequently in patients with a history of HF compared with those without, but both sets of participants experienced comparable reductions in the risks of these outcomes with the use of canagliflozin. Labeled adverse effects of canagliflozin on amputation and fracture were comparable among patients with and without HF at baseline, but there were possibly lower absolute risks of adverse events related to osmotic diuresis among patients with HF. There was no statistical evidence that adverse events attributable to volume depletion or acute kidney injury were differentially increased by treatment with canagliflozin in those with HF compared with those without HF, although CIs about estimates were wide.

The benefits for HF outcomes appeared early during follow-up, suggesting a mode of action driven primarily by volume and hemodynamic effects. Reductions in preload and afterload stemming from natriuresis,^14^ systemic blood pressure lowering,^17^ modification of the intrarenal renin angiotensin axis,^18^ and reduction in arterial stiffness^19^ may all contribute to the protection afforded. Preservation of renal function and the mitigation of volume overload achieved with SGLT2 inhibition also probably contributed to the observed reduction in HF risk. By contrast, antiatherosclerotic effects of SGLT2 inhibition mediated through effects on glucose, blood pressure, and obesity are unlikely to have played a major role in the large and early benefit observed for this outcome.

There may also be direct positive effects of SGLT2 inhibition on cardiac metabolism that are attributable to a shift from fatty acids to ketone bodies as the substrate for myocardial energy generation. Metabolic studies have shown that the hypertrophied and failing heart uses ketone bodies as an alternate fuel source,^20,21^ and increased hepatic neogenesis of ketone bodies is an established effect of SGLT2 inhibitors.^22,23^ Enhanced cardiac efficiency may also be facilitated by increased oxygen delivery resulting from SGLT2 inhibitor–associated hemoconcentration.^18^ Although the SGLT2 receptor is expressed primarily on the luminal surface of the proximal tubule in the kidney, there has been 1 report of SGLT2 expression in heart tissue.^24^

The findings reported here are strengthened by the rigorous design and conduct of the trial, the prespecification of HF as an outcome of interest, and the careful masked adjudication of all relevant events by an expert committee. Capturing the different modes of HF death as a separate cause-specific outcome is challenging and may underestimate the fatal disease burden attributable to HF. Accordingly, we selected the composite of cardiovascular death and hospitalized HF as the primary outcome because of its clinical relevance while also reporting on other more narrowly defined outcomes incorporating events explicitly defined as HF death. The relatively few primary outcome events recorded limits the capacity to detect effects and makes difficult interpretation of borderline significant findings (eg, the interactions of canagliflozin treatment and HF prevention with baseline characteristics, such as obesity and use of some drug therapies). Interpretation is further complicated by the overlap in these baseline characteristics across participant subgroups. The limited documentation of HF at baseline, and specifically the absence of systematically collected baseline biomarkers or echocardiography data, meant that the estimated prevalence of established HF was imperfect and there was likely some misclassification of patients according to the presence or absence of HF at baseline. It was also not possible to classify baseline HF according to preservation or reduction in ejection fraction. The low rates of loop diuretic use among patients with HF at baseline suggests that most had nonsevere disease and raises additional uncertainty about the HF diagnoses at baseline in some patients.

The effects on HF observed within the CANVAS Program appear mostly comparable to those reported for the EMPA-REG OUTCOME trial. An exception was the observation of a borderline significant greater proportional risk reduction for individuals with a history of HF at baseline in the CANVAS Program, which was not matched by a corresponding finding in the analyses of the EMPA-REG OUTCOME trial. This might reflect the different characteristics of the included populations or the slightly different criteria used to define HF outcomes between the 2 studies. However, the multiple and post hoc analyses of HF done for the CANVAS Program and EMPA-REG OUTCOME had limited statistical power to test for interactions, and the risk of missing real differences or observing spurious chance differences is high.

The CANVAS Program data provide clear evidence of the protective effects of canagliflozin on HF and, in conjunction with EMPA-REG OUTCOME, suggest an important role for SGLT2 inhibitors in the prevention of HF among patients with type 2 diabetes mellitus. Additional data from ongoing trials in diabetes mellitus will further clarify the impact of SGLT2 inhibitors on this major cause of mortality and morbidity^25,26^ and confirm or refute hypotheses raised by the CANVAS and EMPA-REG OUTCOME trial findings. A series of new trials specifically exploring mechanisms and testing effects on HF outcomes among patients without diabetes mellitus^27–30^ will also provide further insight into the mode of action by which benefits are achieved. In conclusion, among patients with type 2 diabetes mellitus and an elevated risk of cardiovascular disease, canagliflozin reduced the risk of cardiovascular death or hospitalized HF across a broad range of different patient groups and in addition to concomitant therapies for HF. Benefits may be greater in patients with a baseline history of HF compared with those without a history of HF.

## Acknowledgments

This study was supported by Janssen Research & Development, LLC. The authors thank all investigators, study teams, and patients for participating in these studies. The authors thank the following people for their contributions to the statistical monitoring/analyses and the protocol development, safety monitoring, and operational implementation over the duration of both studies: Lyndal Hones, Lucy Perry, Sharon Dunkley, Qiang Li, Severine Bompoint, Laurent Billot, Mary Lee, Joan Lind, Roger Simpson, Mary Kavalam, Frank Vercruysse, Elisa Fabbrini, Richard Oh, Ngozi Erondu, and Norm Rosenthal. Medical writing support was provided by Kimberly Dittmar, PhD, of MedErgy, and was funded by Janssen Global Services, LLC. Canagliflozin has been developed by Janssen Research & Development, LLC, in collaboration with Mitsubishi Tanabe Pharma Corporation.

## Sources of Funding

This study was supported by Janssen Research & Development, LLC.

## Disclosures

Dr Rådholm reports receiving funding from a County Council of Östergötland International Fellowship. Dr Figtree reports receiving research support from the cofunded National Health and Medical Research Council and Heart Foundation (Australia) Fellowship and the Heart Research Australia, and compensation from Janssen for serving on the Adjudication Panel of the CANVAS Program. Dr Perkovic reports receiving research support from the Australian National Health and Medical Research Council (Senior Research Fellowship and Program Grant); serving on Steering Committees for AbbVie, Boehringer Ingelheim, Glaxo SmithKline, Janssen, Novartis, and Pfizer; and serving on advisory boards and speaking at scientific meetings for AbbVie, Astellas, AstraZeneca, Baxter, Bayer, Bristol-Myers Squibb, Boehringer Ingelheim, Durect, Eli Lilly, Gilead, Glaxo SmithKline, Janssen, Merck, Novartis, Novo Nordisk, Pfizer, Pharmalink, Relypsa, Retrophin, Roche, Sanofi, Servier, and Vitae. Dr Solomon reports having received compensation from Janssen for serving on the DSMB of the CANVAS trial. Outside the scope of this study, Dr Solomon has received research grants from Alnylam, Amgen, AstraZeneca, Bellerophon, Celladon, Gilead, GlaxoSmithKline, Ionis Pharmaceutics, Lone Star Heart, Mesoblast, MyoKardia, National Institutes of Health/National Heart, Lung, and Blood Institute, Novartis, Sanofi Pasteur, and Theracos; and has consulted for Alnylam, Amgen, AstraZeneca, Bayer, BMS, Corvia, Gilead, GSK, Ironwood, Merck, Novartis, Pfizer, Takeda, and Theracos. Dr Mahaffey’s financial disclosures can be viewed at http://med.stanford.edu/profiles/kenneth-mahaffey. Dr de Zeeuw reports serving on advisory boards and speaking for Bayer, Boehringer Ingelheim, Eli Lilly, Fresenius, and Mitsubishi-Tanabe; Steering Committees and speaking for Abb Vie, Astellas, and Janssen; and Data Safety and Monitoring Committees for Bayer, with all honoraria paid to his institution. Dr Fulcher reports receiving research support from Novo Nordisk and serving on advisory boards and as a consultant for Boehringer Ingelheim, Dohme, Janssen, Merck Sharp, and Novo Nordisk. Drs Barrett, Shaw, and Desai report being full-time employees of Janssen Research & Development, LLC. Dr Matthews reports receiving research support from Janssen; serving on advisory boards and as a consultant for Eli Lilly, Janssen, Novartis, Novo Nordisk, Sanofi-Aventis, and Servier; and giving lectures for Aché Laboratories, Eli Lilly, Janssen, Mitsubishi Tanabe, Novartis, Novo Nordisk, Sanofi-Aventis, and Servier. Dr Neal reports receiving research support from the Australian National Health and Medical Research Council Principal Research Fellowship and Janssen; and serving on advisory boards and being involved in CME programs for Janssen, with any consultancy, honoraria, or travel support paid to his institution. He notes institutional relationships with AbbVie, Actelion, and Janssen.

## Supplementary Material
