# Supplementary material for: Canagliflozin and Heart Failure in Type 2 Diabetes Mellitus: Results From the CANVAS Program
Source: Circulation. 2018 Jul 30;138(5):458–68. doi: 10.1161/CIRCULATIONAHA.118.034222 (PMC6075881; doi:10.1161/CIRCULATIONAHA.118.034222)
Supplement: Supplementary file 1 [file cir-138-458-s001.pdf]

SUPPLEMENTAL MATERIAL

Supplemental Appendix 1. CANVAS Program sites and investigators

CANVAS

Argentina: Pablo Arias, Maria Rosa Ulla, Andres Alvarisqueta, Laura Maffei, Jose Osvaldo Fretes, Silvia Gorban De Lapertosa, Virginia Visco, Georgina Sposetti, Javier Farias, Eduardo Francisco Farias, Maria Cecilia Cantero, Rodolfo Feldman, Maria Carolina Ridruejo, Pedro Calella, Cesar Zaidman; **Australia:** Stephen Stranks, Peak Man Mah, Alison Nankervis, Duncan Topliss, Georgia Soldatos, Richard Simpson, Murray Gerstman, David Colquhoun, Ferdinandus De Looze, Robert Moses, Michael Suranyi, Samantha Hocking, David Packham, Duncan Cooke, Karam Kostner; **Belgium:** Eric Weber, Chris Vercammen, Luc Van Gaal, Jozef Tits, Bart Keymeulen, Chantal Mathieu; **Canada:** Naresh Aggarwal, Dan Dattani, Francois Blouin, Richard Dumas, Sam Henein, Patrick Ma, Ali Najarali, Michael Omahony, Tracy Pella, Wilson Rodger, Daniel Shu, Vincent Woo, Brian Zidel, Lew Pliamm, Brian Ramjattan, Ronald Akhras, Jasmin Belle-Isle, Stuart Ross, Geza Molnar; **Colombia:** Juan Manual Arteaga, Ivonne Jarava; **Czech Republic:** Alena Andresova, Miloslava Komrskova, Cyril Mucha, Tomas Brychta, Dagmar Bartaskova, Romana Urbanova, Tomas Spousta, Jana Havelkova, Tomas Sedlacek, Milan Kvapil; **Estonia:** Ülle Jakovlev, Verner Fogel, Liina Viitas, Mai Soots, Maire Lubi, Marju Past, Jelena Krasnopejeva; **Germany:** Hasan Alawi, Klaus Busch, Felix Klemens Pröpper, Andrea Thron, Stephan Jacob, Andreas Pfützner, Ludger Rose, Thomas Segiet, Christine Kosch, Andrea Moelle; **Great Britain:** Melanie Davies, Hamish Courtney, Martin Gibson, Luigi Gnudi, Frances Game, John Wilding, Thozhukat Sathyapalan, Miles Fisher, Shenaz Ramtoola, Satyan Rajbhandari, Maurice Okane; **Hungary:** Eleonora Beke, Ferenc Poor, Karoly Nagy, Gyozo Kocsis, Tamas Oroszlan, Peter Faludi, Mihaly Gurzo; **India:** Sathyanarayana Srikanta, Mala Dharmalingam, Bala Murugan, Pramod Gandhi, Bipin Sethi, Sosale Aravind, Sharda Ardhanareeshwaran, Arpan Bhattacharyya, Ganapathi Bantwal, Vijay Viswanathan, Paramesh Shamanna, Banshi Saboo, Viswanathan Mohan, Reshma Parmaj, Kirti Kumar Modi, Sindhu Joshi, Sunil Jain, Sanjay Kalra, Arun Chankramath Somasekharan, Prabha Adhikari, Ajay Kumar, Harshada Kudalkar, Rajiv Passey, Mathew John, Sadasivarao Yalamanchi, Keyur Parikh, K.P. Rajesh, Rajesh Nair, Ajay Kumar, Sasi Kumar, Lily Rodrigues, Pawan Gangwal, Pankaj Agarwal, Sandeep Kumar Gupta, Abhay Amrutlal Mutha, Shailaja Dilip Kale, Ravindra Laxman Kulkarni, Sandip Chudasama, Kamal Sharma, Anoop Nambiar, Aniruddha Tangaonkar, Vaishali Deshmukh, Biswakesh Majumdar, Rajendran Veerappan, Deepak Namjoshi; **Israel:** Itamar Raz, Julio Weinstein, Ilana Harman Boehm, Victor Vishlitzky; **Luxembourg:** Frederic Dadoun; **Malaysia:** Rajesh P. Shah, Lai Seong Hooi, Alexander Tan, Wan Mohamad Wan Bebakar, Mafauzy Mohamed, Amir S. Khir, Norlela Sukor, Khalid Abdul Kadir; **Mexico:** Enrique Morales, Sergio Zuñiga, Melchor Alpizar, Cesar Calvo, Rolando Zamarripa, Juan Rosas, Armando Vargas; **The Netherlands:** Max Nieuwdorp, Vicdan Kose, Susanne Kentgens, Gloria Rojas, Wouter Van Kempen, Jacqueline Hoogendijk, Mazin Alhakim, Victor Gerdes, Marcel Hovens, Johan Berends, A. Woittiez, Cees Jan Smit, B. Dekkers, Wilco Spiering, Marcel K. Van Dijk-Okla, Ben P.M. Imholz, Ruud J.M. Van Leendert, Marije Ten Wolde, Peter J.H. Smak Gregoor; **New Zealand:** Russell Scott, Jeremy Krebs, John Baker, Joe Singh, Calum Young; **Norway**: Gisle Langslet, Hans Olav Hoivik, Torbjorn Kjaernli, Sigbjorn Elle, Eric Gjertsen, Knut Risberg, Andreas Tandberg, Leidulv Solnoer, Per Anton Sirnes; **Poland:** Tadeusz Derezinski, Malgorzata Arciszewska, Edward Franek, Ewa Szyprowska, Dariusz Sowinski,

Robert Petryka, Beata Czakanska-Dec, Grazyna Pulka, Katarzyna Jusiak, Mariusz Dabrowski, Piotr Kubalski, Malgorzata Wojciechowska, Andrzej Madej, Danuta Pupek-Musialik; **Russia:** Natalia Blinova, Ludmila Kondratjeva, Anatoly Kuzin, Mikhail Boyarkin, Tatyana Gomova, Alexander Khokhlov, Sergey Vorobjev, Olga Mirolyubova, Svetlana Boldueva, Olga Ershova, Marina Ballyzek, Olga Smolenskaya, Sergey S. Yakushin, Dmitry Zateyshchikov, Mikhail Arkhipov, Alexandr Kuzmenko, Ivan Maksimov, Igor Motylev, Vladimir Rafalskiy, Leonid Strongin, Tatyana Treshkur, Natalya Volkova, Olga Barbarash, Tatiana Raskina, Leonid Bartosh, Inna Nikolskaya, Elena Shutemova, Viktor Gurevich, Natalia Burova, Elena Vorobyeva, Denis Andreev, Boris Bart, Tatiana Khlevchuk, Lyudmila Gapon, Ivan Gordeev, Nikolai Gratsiansky, Alsu Zalevskaya, Sergey Sayganov, Oleg Solovyev, Galina Reshedko, Natalia Shilkina, Petr Chizhov, Julia Shapovalova, Alexander Sherenkov, Olga Reshetko, Vladimir Simanenkov; **Spain:** Juan Garcia Puig, Jose Saban, Jose Pascual, Jose Dominguez, Elias Delgado, Carlos Calvo, Manuel Vida, Santiago Duran, Francisco Tinahones, Jordi Salas, Jose Miguel Gonzalez, Manuel Monreal, Armand Grau, Andreu Nubiola, Pere Alvarez; **Sweden:** Kaj Stenlöf, Pekka Koskinen, Carl-Johan Lindholm, Ulrik Mathiesen, Katarina Berndtsson Blom, Bengt-Olov Tengmark, Hans Jul-Nielsen; **Ukraine:** Oleksandr Larin, Svetlana Panina, Svitlana Kovalenko, Olena Voloshyna, Vera Tseluyko, Olga Gyrina, Vadim Vizir, Olga Barna, Maryna Dolzhenko, Yuriy Mostovoy, Vadim Korpachev, Boris Mankovskiy, Mykola Vatutin; **United States:** Charles Arena, Basil Akpunonu, Rahfa Zerikly, Claire Baker, Toby Briskin, Darlene Bartilucci, Joshua Barzilay, Christian Breton, John Buse, Richard Cherlin, Michael Cobble, Clarence Ellis, Raymond Fink, Alan Forker, Ronald Garcia, Priscilla Hollander, Angela House, Daniel Hyman, Richard Ingebretsen, David Jack, Judith Kirstein, Kerri Kissell, Daniel Lorber, Donald McNeil, Wendell Miers, Alex Murray, Robert Call, Stephen T. Ong, Fernando Ovalle, Robert Pearlstein, Veronica Piziak, Daniel Pomposini, David Robertson, Julio Rosenstock, Ulrich Schubart, Shaukat Shah, Rodney Stout, Mark Turner, James Wallace, Leonard Chuck, Edmund Claxton, Emily Morawski, Alan Wynne, Carol Wysham, Michael Alderman, Walter Patton, Bryan Pogue, Arnold Silva, Roger Guthrie, Sam Lerman, Robert Madder, Wendy Miller, Daniel Weiss, Dean Kereiakes, Ronald J Graf, Negah Rassouli, James Greenwald, Hanna Abu-Nassar, Derek Muse, Vicki Kalen, Natalia Hegedosh, Richard Dobrusin, Glover Johnson, Tami Bruce, Gary Gleason.

CANVAS-R

Argentina: Marisa Vico, Sonia Hermida, Lucrecia Nardone, Laura Maffei, Javier Farias, Elizabeth Gelersztein, Maximiliano Sicer, Andres Alvarisqueta, Georgina Sposetti, Virginia Visco, Rodolfo Feldman, Silvia Orio; **Australia:** Christopher Nolan, Michael Suranyi, Samantha Hocking, Stephen Stranks, Duncan Cooke, Ferdinandus de Looze, Ashim Sinha, Timothy Davis, Anthony Russell, Acharya Shamasunder, Murray Gerstman, Richard MacIsaac; **Belgium:** Chris Vercammen, Luc Van Gaal, Chantal Mathieu, Xavier Warling, Jan Behets, Andre Scheen, Guy T'Sjoen, Ann Verhaegen, Isabelle Dumont, Youri Taes, Francis Duyck, Fabienne Lienart; **Brazil:** Adolfo Sparenberg, Adriana Costa e Forti, Andressa Leitao, Cariolina Jungers di Siqueira Chrisman, César Hayashida, Daniel Panarotto, Fabio Rossi dos Sanos, Fadlo Fraige Filho, Flávia Coimbra Maia, Gilmar Reis, Hugo Lisboa, Joao Felicio, Joselita Siqueira, Lilia Nigro Maia, Luiz Alberto Andreotti Turatti, Maria José Cerqueira, Maria Tereza Zanella, Patricia Muszkat, Miguel Nasser Hissa, Teresa Bonansea; **Canada:** Igor Wilderman, Vincent Woo, Richard Dumas, Francois Blouin, Pierre Filteau, George Tsoukas, Peter Milne, Dan Dattani, Chantal Godin, Michael Omahony, Daniel Shu, Jasmin Belle-Isle, Douglas Friars, Anil

Gupta, Ted Nemtean, Andrew Steele; **China:** Zhan-Quan Li, Changsheng Ma, Linong Ji, Shuguang Pang, Yan Jing, Ruiping Zhao, Ruifang Bu; **Czech Republic:** Tomas Spousta, Tatana Souckova, Dagmar Bartaskova, Pavlina Kyselova, Lea Raclavska, Milan Kvapil, Jana Havelkova, Emilia Malicherova; **France:** Philippe Zaoui, Didier Gouet, Jean-Pierre Courreges, Salha Fendri, Samy Hadjadj, Bruno Verges, Bogdan Nicolescu Catargi, Sylvaine Clavel, Jean-Jacques Altman, Agnes Hartemann, Gaétan Prevost; **Germany:** Diethelm Tschöpe, Elena Henkel, Rolf Göbel, Jochen Seufert, Hermann Haller, Thomas Behnke, Andreas Pfützner, Gerhard Klausmann, Klaus Busch, Baerbel Hirschhaeuser, Stephan Jacob; **Great Britain:** Melanie Davies, Rob Andrews, Narayan Annamalai, Hamish Courtney, Srikanth Bellary, Mark Blagden, John Clark, Steven Creely, Ken Darzy, Iskandar Idris, Richard Falk, Lucinda Summers, Njaimeh Asamoah, Andrew Johnson, See Kwok, Shenaz Ramtoola, Gerry Rayman, Jamie Smith, John Wilding; **Hungary:** Marietta Baranyai, Katalin Csomos, Mihaly Gurzo, Eleonóra Harcsa, Nikosz Kanakaridisz, Nóra Késmárki, Tamas Oroszlan, József Pátkay, Eva Peterfai, Balázs Gaszner, Ildiko Jozsef; **Italy:** Stefano Genovese, Antonio Ettore Pontiroli, Enzo Bonora, Dario Giugliano, Domenico Cucinotta, Giorgio Sesti, Paola Ponzani, Giuseppe Pugliese, Giulio Marchesini Reggiani, Paolo Pozzilli, Sergio Leotta, Emanuela Orsi, Carlo Giorda, Paolo Di Bartolo; **Korea:** Tae-Sun Park, Chung-Gu Cho, In-Joo Kim, Il Seong Nam-Goong, Choon Hee Chung, Ho Chan Cho, Dong-Seop Choi, Kun-Ho Yoon, Nan-Hee Kim, Kyung-Mook Choi, Kyu-Jeung Ahn, Ji-Oh Mok, Soon-Jib Yoo, Tae-Keun Oh, Kwan-Woo Lee, Hak-Chul Jang, Jeong-Hyun Park, In-Kyu Lee, Byung-Joon Kim, Doo-Man Kim, Ho Sang Shon, Moon-Kyu Lee, ShinGon Kim; **Malaysia:** Mafauzy Mohamed, Paranthaman Vengadasalam, Alexander Tong Boon Tan, Wan Mohd Izani Wan Mohamed, Rajesh P Shah, Khalid Yusoff, Amir Sharifuddin Mohd Khir, Florence Tan, Mansor Yahya; **Mexico:** Rafael Violante, Manuel Odin De los Rios, Marco Alcocer, Enrique Morales, Juan Rosas, Armando Vargas, Manuel González, Esperanza Martinez, Jorge Antonio Aldrete, Guillermo Gonzalez, Cynthia Mustieles Rocha, Leobardo Sauque, Paul Frenk, José Luis Arenas; **The Netherlands:** Peter Tichelaar, A Kooy, Albert Van de Wiel, Gerben Lochorn, Peter De Vries, Hans Feenstra, Max Nieuwdorp, Wouter Van Kempen, Mazin Alhakim, Ben Imholz, Ruud van Leendert, Peter Smak Gregoor, Joop Brussen, Hanno Pijl, Manuel Castro Cabezas, F Gonkel, P Smits, Daan Lansdorp, Susanne Kentgens, Aletha Veenendaal, Gloria Rojas; **New Zealand:** John Richmond, Russell Scott, Mike Williams, Dean Quinn, Jeremy Krebs, John Baker, Veronica Crawford, Calum Young; **Poland:** Malgorzata Arciszewska, Krystyna Jedynasty, Dariusz Sowinski, Ewa Szyprowska, Andrzej Madej, Miroslawa Polaszewska-Muszynska, Danuta Zytkiewicz-Jaruga, Katarzyna Wasilewska, Piotr Romanczuk, Anna Ocicka-Kozakiewicz, Czeslaw Marcisz, Boguslaw Okopien, Anna Bochenek, Lukasz Wojnowski, Teresa Sliwinska, Barbara Rewerska, Witold Zmuda, Katarzyna Klodawska, Ewa Skokowska, Jacek Fabisiak, Cezary Danilkiewicz; **Puerto Rico**: Elba Perez Vargas, Elizabeth Barranco Santana; **Russia**: Tatiana Raskina, Olga Barbarash, Leonid Bartosh, Igor Motylev, A Kuzin, Olga Reshetko, Tatyana Zykova, Olga Ershova, Marina Balyzek, Vladimir Rafalsky, Natalya Volkova, Nina Nosova, Natalia Burova, Alsu Zalevskaya, Galina Reshedko, Natalia Shilkina, Petr Chizhov, Alexander Sherenkov, Vladimir Simanenkov, Tatiana Lysenko, Irina Ipatko, Mikhail Boyarkin, Sergey Vorobyev, Lyudmila Gapon, Andrey Obrezan, Valeria Esip, Zhanna Paltsman, Andrey Verbovoy, Fatima Khetagurova, Yuri Shvarts; **Spain:** Pere Alvarez-Garcia, Francisco Martinez Deben, Josep M Grinyo, Carlos Calvo, Carmen Suarez, JM Pascual, Jose Dominguez, Anna Oliveras, Armand Grau, Fernando Gómez Peralta, Luis Alvarez-Sala, Cañizo Francisco, Jorge Gómez Cerezo, Juan Garcia Puig, Carlos Trescolí, Francisco Jose Fuentes Jimenez, Santiago Tofé, Judith López, Javier Nieto Iglesias, Luis Vigil, Santiago Duran

Garcia, Jose Luis Gorriz, Pilar Saavedra Vallejo, Francisco Tinahones Madueno, Jose Luis Blanco Coronado, Alfonso Soto, Luis De Teresa, Jose Miguel Gonzalez, Antonio Rodriguez Botaro, Carmina Cuesta; **Sweden:** Bjorn Bragée, Bengt-Olov Tengmark, Hans Jul-Nielsen, Pekka Koskinen, Linda Moris, Fredrik Huss, Pär Jennersjö, Katarina Berndtsson-Blom, Bo Liu, Kaj Stenlöf, Carl-Johan Lindholm, Johan Jendle; **Taiwan:** Dee Pei, Wayne H-H Shue, Chern-En Chiang, Ching-Chu Chen, Ming-Nan Chien, Ping-Yen Liu, Ching-Ling Lin, Yi-Jing Sheen; **Ukraine:** Dmytro Reshotko, Nikolay Rishko, Olexander Samoylov, Valentina Serkova, Ivan Smirnov, Liubov Sokolova, Vira Tseluyko, Vadym Vizir, Tetiana Zlova, Vitaliy Maslyanko, Oleksandr Larin, Valentina Velichko, Lyudmila Prystupa, Nadiya Yarema, Galina Mishanich, Iryna Bondarets, Nataliya Virstyuk, Olexander Serhiyenko, Stepan Pavlyk, Olena Levchenko, Orest Abrahamovych, Volodymyr Botsurko, Maryna Dolzhenko, Victoria Chernikova, Yuriy Karachentsev, Vitaliy Katerenchuk, Vadym Korpachov, Yaroslav Malynovsky, Boris Mankovsky, Yuriy Mostovoy, Larisa Pererva, Nataliya Pertseva; **United States:** Vicki Conrad, Kenneth Fox, David Jack, Robert Buynak, Michael Dever, John Kirby, Larry Odekirk, Priyantha Wijewardane, Robert Carson, Bruce Seaton, Ann Elizabeth Mohart, Salvatore Bianco, Michael R Cox, Andrew Kim, Steven Geller, Jakkidi Reddy, Derek Muse, Alan Wynne, Harold Bays, Judith Kirstein, James Riser, Ahmed Arif, Claire Baker, Kim Barbel-Johnson, Gary Bedel, Pierre Blemur, Christian Breton, Anna Chang, Brian Naccari, Nancy Jo Coburn, Lisa Cohen, Eric Dedeke, Charles Diederich, John Earl, Anu George, Matthew Gilbert, Gary Gleason, Gregory Haase, Rodney Ison, Mahendra Jain, Imtiaz Alam, Sam Lerman, Lawrence Levinson, Lon D Lynn, Michael Oliver, Barry Kusnick, Robert Pearlstein, Sanford Plevin, Samuel Mujica Trenche, Vernon Young, Michael Jutovsky, Ralph Wade, James Wallace, Albert Weisbrot, Duane Wombolt, Alan Forker, Jalal Taslimi, Roger Guthrie.

Supplemental Appendix 2. CANVAS Program committees

Steering Committee

David R. Matthews (Co-chair), Bruce Neal (Co-chair), Greg Fulcher, Kenneth W. Mahaffey, Vlado Perkovic, Mehul Desai (Sponsor), Dick de Zeeuw

Independent Data Monitoring Committee

Philip Home (Chair), Jeffrey L. Anderson, Ian W. Campbell, John Lachin (withdrew in September 2015), Daniel Scharfstein, Scott D. Solomon, Robert G. Uzzo

Cardiovascular Adjudication Committee

Greg Fulcher (Chair), John Amerena, Clara Chow, Gemma Figtree, John French, Graham Hillis, Mark A. Hlatky, Bronwyn Jenkins, Nicholas J. Leeper, Richard Lindley, Barry McGrath, Alison Street, John Watson

Renal Adjudication Committee

Greg Fulcher (Chair), Shahnaz Shahinfar, Tara Chang, Arjun D. Sinha, Phyllis August

Safety Adjudication

Fracture Adjudication: Bioclinica

Diabetic Ketoacidosis Adjudication: Baim Institute for Clinical Research

Pancreatitis Adjudication Committee: Adam Cheifetz (Chair), Sunil Sheth, Joseph Feuerstein

Supplemental Appendix 3. CANVAS Program cardiovascular death and heart failure criteria

Definition of Cardiovascular Death

Cardiovascular death includes death resulting from an acute MI, sudden cardiac death, death due to heart failure, death due to stroke, and death due to other cardiovascular causes, as follows:

1. **Death Due to Acute MI** refers to a death by any mechanism (arrhythmia, heart failure [HF], low output) within 30 days after a MI related to the immediate consequences of the myocardial infarction, such as progressive congestive heart failure (CHF), inadequate cardiac output, or recalcitrant arrhythmia. If these events occur after a "break" (e.g., a CHF- and arrhythmia-free period of at least a week), they should be designated by the immediate cause, even though the MI may have increased the risk of that event (e.g., late arrhythmic death becomes more likely after an acute MI). The acute MI should be verified to the extent possible by the diagnostic criteria outlined for acute MI or by autopsy findings showing recent MI or recent coronary thrombus. Sudden cardiac death, if accompanied by symptoms suggestive of myocardial ischemia, new ST elevation, new left bundle branch block (LBBB), or evidence of fresh thrombus by coronary angiography and/or at autopsy should be considered death resulting from an acute MI, even if death occurs before blood samples or 12-lead electrocardiogram (ECG) could be obtained, or at a time before the appearance of cardiac biomarkers in the blood. Death resulting from a procedure to treat a MI (percutaneous coronary intervention [PCI], coronary artery bypass graft surgery [CABG]), or to treat a complication resulting from MI, should also be considered death due to acute MI.

   Death resulting from a procedure to treat myocardial ischemia (angina) or death due to a MI that occurs as a direct consequence of a cardiovascular investigation/procedure/operation should be considered as a death due to other cardiovascular causes.

2. **Sudden Cardiac Death** refers to a death that occurs unexpectedly, not following an acute MI, and includes the following deaths:
   a. Death witnessed and instantaneous without new or worsening symptoms
   b. Death witnessed within 60 minutes of the onset of new or worsening cardiac symptoms, unless the symptoms suggest acute MI
   c. Death witnessed and attributed to an identified arrhythmia (e.g., captured on an ECG recording, witnessed on a monitor, or unwitnessed but found on implantable cardioverter-defibrillator review)
   d. Death after unsuccessful resuscitation from cardiac arrest
   e. Death after successful resuscitation from cardiac arrest and without identification of a noncardiac etiology (postcardiac arrest syndrome)
   f. Unwitnessed death without other cause of death (information regarding the patient's clinical status preceding death should be provided, if available)

General Considerations

- A subject seen alive and clinically stable 12-24 hours prior to being found dead without any evidence or information of a specific cause of death should be classified as "sudden cardiac death." Typical scenarios include:
  - Subject well the previous day but found dead in bed the next day
  - Subject found dead at home on the couch with the television on
- Deaths for which there is no information beyond "Patient found dead at home" may be classified as "death due to other cardiovascular causes" or in some trials, "undetermined cause of death." Please see *Definition of Undetermined Cause of Death*, for full details.

3. **Death Due to HF or Cardiogenic Shock** refers to a death occurring in the context of clinically worsening symptoms and/or signs of heart failure without evidence of another cause of death and not following an acute MI. Note that deaths due to HF can have various etiologies, including one or more acute MIs (late effect), ischemic or nonischemic cardiomyopathy, or valve disease.

Death due to HF or Cardiogenic Shock should include sudden death occurring during an admission for worsening heart failure as well as death from progressive HF or cardiogenic shock following implantation of a mechanical-assist device.

New or worsening signs and/or symptoms of CHF include any of the following:

a. New or increasing symptoms and/or signs of HF requiring the initiation of, or an increase in, treatment directed at HF or occurring in a patient already receiving maximal therapy for HF
b. HF symptoms or signs requiring continuous intravenous therapy or chronic oxygen administration for hypoxia due to pulmonary edema
c. Confinement to bed predominantly due to HF symptoms
d. Pulmonary edema sufficient to cause tachypnea and distress **not** occurring in the context of an acute MI, worsening renal function, or as the consequence of an arrhythmia occurring in the absence of worsening heart failure
e. Cardiogenic shock **not** occurring in the context of an acute MI or as the consequence of an arrhythmia occurring in the absence of worsening HF

Cardiogenic shock is defined as systolic blood pressure (SBP) <90 mmHg for greater than 1 hour, not responsive to fluid resuscitation and/or heart rate correction, and felt to be secondary to cardiac dysfunction and associated with at least one of the following signs of hypoperfusion:

- Cool, clammy skin ***or***
- Oliguria (urine output <30 ml/hour) ***or***
- Altered sensorium ***or***
- Cardiac index <2.2 $l/min/m^2$

Cardiogenic shock can also be defined if SBP <90 mmHg and increases to ≥90 mmHg in less than 1 hour with positive inotropic or vasopressor agents alone and/or with mechanical support.

General Considerations

HF may have a number of underlying causes, including acute or chronic ischemia, structural heart disease (e.g., hypertrophic cardiomyopathy), and valvular heart disease. Where treatments are likely to have specific effects, and it is likely to be possible to distinguish between the various causes, then it may be reasonable to separate out the relevant treatment effects. For example, obesity drugs such as fenfluramine (pondimin) and dexfenfluramine (redux) were found to be associated with the development of valvular heart disease and pulmonary hypertension. In other cases, the aggregation implied by the definition above may be more appropriate.

4. **Death Due to Stroke** refers to death occurring up to 30 days after a stroke that is either due to the stroke or caused by a complication of the stroke.

5. **Death Due to Other Cardiovascular Causes** refers to a cardiovascular death not included in the above categories (e.g., dysrhythmia unrelated to sudden cardiac death, pulmonary embolism, cardiovascular intervention [other than one related to an acute MI], aortic aneurysm rupture, or peripheral arterial disease). Mortal complications of cardiac surgery or nonsurgical revascularization should be classified as cardiovascular deaths.

Hospitalized Congestive Heart Failure

HF requiring hospitalization is defined as an event that meets the following criteria:

1. Requires hospitalization defined as an admission to an inpatient unit or a visit to an emergency department that results in at least a 24-hour stay (or a date change if the time of admission/discharge is not available).
   AND
2. Clinical symptoms of HF, including ≥1 of the following new or worsening conditions:
   - Dyspnea
   - Orthopnea
   - Paroxysmal nocturnal dyspnea
   - Increasing fatigue/worsening exercise tolerance

   AND
3. Physical signs of HF, including ≥2 of the following:
   - Edema (greater than 2+ lower extremity)
   - Pulmonary crackles greater than basilar (pulmonary edema must be sufficient to cause tachypnea and distress not occurring in the context of an acute MI or as the consequence of an arrhythmia occurring in the absence of worsening HF)
   - Jugular venous distension
   - Tachypnea (respiratory rate >20 breaths/minute)
   - Rapid weight gain
   - S3 gallop
   - Increasing abdominal distension or ascites
   - Hepatojugular reflux
   - Radiological evidence of worsening HF
   - A right heart catheterization within 24 hours of admission showing a pulmonary

capillary wedge pressure (pulmonary artery occlusion pressure) ≥18 mmHg or a cardiac output <2.2 $l/min/m^2$

Note: biomarker results (e.g., brain natriuretic peptide [BNP]) consistent with CHF will be supportive of this diagnosis, but the elevation in BNP cannot be due to other conditions such as cor pulmonale, pulmonary embolus, primary pulmonary hypertension, or congenital heart disease. Increasing levels of BNP, although not exceeding the ULN, may also be supportive of the diagnosis of CHF in selected cases (e.g., morbid obesity).

AND

4. Need for additional/increased therapy
   - Initiation of, or an increase in, treatment directed at HF or occurring in a patient already receiving maximal therapy for HF and including ≥1 of the following:
     - Initiation of or a significant augmentation in oral therapy for the treatment of CHF
     - Initiation of intravenous diuretic, inotrope, or vasodilator therapy
     - Up-titration of intravenous therapy, if already on therapy
     - Initiation of mechanical or surgical intervention (mechanical circulatory support, heart transplantation or ventricular pacing to improve cardiac function), or the use of ultrafiltration, hemofiltration, or dialysis that is specifically directed at treatment of HF.

AND

5. No other noncardiac etiology (such as chronic obstructive pulmonary disease, hepatic cirrhosis, acute renal failure, or venous insufficiency) and no other cardiac etiology (such as pulmonary embolus, cor pulmonale, primary pulmonary hypertension, or congenital heart disease) for signs or symptoms is identified.

Note: it is recognized that some patients may have multiple simultaneous disease processes. Nevertheless, for the endpoint event of HF requiring hospitalization, the diagnosis of CHF would need to be the primary disease process accounting for the above signs and symptoms.
